# Nanoparticle-Based DNA Biosensor: Synthesis of Novel Manganese Nanoparticles Applied in the Development of a Sensitive Electrochemical Double-Stranded/Single-Stranded DNA Biosensor

**DOI:** 10.3390/mi16020232

**Published:** 2025-02-18

**Authors:** Dilsat Ozkan-Ariksoysal, Elpida Pantelidou, Catherine Dendrinou-Samara, Stella Girousi

**Affiliations:** 1 Department of Analytical Chemistry, Faculty of Pharmacy, Ege University, Bornova 35100, Izmir, Türkiye; 2 Laboratory of Inorganic Chemistry, School of Chemistry, Aristotle University of Thessaloniki, 541 24 Thessaloniki, Greece; elpipant@gmail.com (E.P.); samkat@chem.auth.gr (C.D.-S.); 3 Laboratory of Analytical Chemistry, School of Chemistry, Aristotle University of Thessaloniki, 541 24 Thessaloniki, Greece

**Keywords:** manganese carbonate nanoparticles, DNA interaction, electrochemical DNA biosensor, pencil graphite electrode, voltammetry

## Abstract

The development of electrochemical DNA biosensors occurred by applying different organically coated Mn-NPs such as MnCO_3_@OAm, MnCO_3_@TEG and MnO_2_/Mn_2_O_3_@TEG, as well as naked MnCO_3_ NPs (where OAm = oleylamine and TEG = tetraethylene glycol). The detection performances of PGEs were modified with different types of Mn-NPs, according to the guanine signal magnitudes obtained after double-stranded DNA (dsDNA) or single-stranded DNA (ssDNA) immobilization at these surfaces. DNA interaction studies were realized using UV-vis, circular dichroism (CD), electrochemical impedance spectroscopy (EIS) and differential pulse voltammetry (DPV) techniques. In addition, a 3- to 5.4-fold increase in guanine response in the presence of dsDNA and a 2.3-fold increase in the presence of ssDNA were obtained with the developed biosensor. The increased signals in DNA immobilization at the electrode surfaces modified with Mn-NPs compared to bare PGE clearly show that the modification of Mn-NPs increases the electroactive surface area of the electrode. The detection limit (LOD) of dsDNA was calculated as 7.86 μg·L^−1^ using the MnO_2_/Mn_2_O_3_@TEG type of the Mn-NP-modified biosensor, while the detection limit of ssDNA was calculated as 3.49 μg·L^−1^ with the MnCO_3_@OAm type Mn-NP-modified biosensor. The proposed sensor was applied to a human DNA sample where the amount of dsDNA extract was found to be 0.62 ± 0.03 mg·L^−1^ after applying the MnO_2_/Mn_2_O_3_@TEG type of Mn-NP-modified biosensor.

## 1. Introduction

The transition metals, of the first row of the periodic table, have not been given much attention in terms of their complex composition, nor have they been particularly studied for their anticancer activity. Among them is the manganese ion, which is involved in various biological systems. It is considered necessary, therefore, to study such systems in terms of geometry, valence, spin, oxidation state and their interaction with DNA.

Manganese-based nanoparticles (Mn-NPs) exhibit unique properties, such as high magnetic susceptibility, catalytic activity, biocompatibility and strong adsorption capacity. Compared with their bulk counterparts, nanosized Mn-NPs are potential adsorbents because of their large surface areas, abundance of defect sites and high surface-to-bulk atom ratios.

In particular, manganese oxide (Mn_x_O_y_) NPs are a versatile class of nanomaterials with a rich array of properties and applications, including materials science, environmental science and medicine [1,2]. They possess high catalytic activity in various chemical reactions, including oxidation and reduction processes [3,4], and high adsorption capacity [5,6]. Their optical properties, including fluorescence and UV–visible absorption, are harnessed in sensing applications, and in combination with their biocompatibility, they can be used in drug delivery and biomedical imaging [7]. Additionally, manganese carbonate (MnCO_3_) is an environmentally friendly material that is found in abundance on Earth and, at the same time, possesses low toxicity and high biocompatibility [8]. Specifically, the low valence (+2) state of MnCO_3_ facilitates the insertion/removal of hydroxyl ions to form MnOHCO_3_ via an electrochemical reaction, leading to manganese-rich valence states (Mn^2+^, Mn^3+^, Mn^4+^, etc.). This property, as well as high electrochemical active centers, rich Faradaic redox process and low cost, makes it a new emerging material for innumerable applications in the field of electrochemical devices and catalysts, such as electrodes for supercapacitors and batteries. The stabilization of the octahedral [MnO_6_] structure due to the carbonate planes in the structure of rhodochrosite can also be beneficial for the occurrence of electrochemical reactions, mainly on the electrode surface, and endow longer cyclic lifetime as a pseudocapacitor [8,9]. Moreover, a study of the interaction of manganese ions with DNA showed that manganese ions bind to the oxygens of the phosphodiester bonds as well as to the DNA bases [10]. At high concentrations of Mn^2+^, DNA precipitation was observed [11,12]. Taking into account the advantages, such as large surface area, good conductivity and excellent biocompatibility, it becomes clear that they offer an environment that is suitable for the immobilization of biological components, while in addition, the high surface-to-volume ratio also implies an increased accumulation of biomolecules.

Electrode surface modification with manganese nanoparticles has emerged as a promising approach to enhance the performance of electrochemical biosensors, including DNA biosensors. Manganese complexes, especially those with redox-active centers, offer catalytic properties that can significantly improve the electron transfer process, making them suitable for electrochemical sensing. In addition, they find applications as modifiers of electrode surfaces in order to electrochemically determine various target substances, while they have also been used as hybridization indicators [13].

The goal, therefore, of using nanomaterials in electrochemical DNA biosensors is to improve the immobilization of DNA molecules and to enhance molecular recognition, but also to enhance the mutation signal with the aim of developing simple and fast analytical methods with high sensitivity and selectivity as well as in vivo analyses.

Εxtensive reviews appeared in the literature showing that many groups of scientists have been involved in the application of nanotechnology to DNA sensors [14,15,16]. But the majority of works refer to complicated and time-consuming electrode modifications that require complex manipulations, thus increasing the cost and the possibility of experimental errors. Although low limits of determination were reached, analyses were performed in these references by applying long experimental modification procedures.

The development of electrochemical DNA biosensors using manganese-based nanoparticles offers an efficient and innovative approach to improve the performance of biosensors. Manganese complexes provide superior catalytic properties, stability and cost-effectiveness, making them ideal candidates for a range of applications in diagnostics, environmental monitoring and food safety [17].

The continued research and optimization of these systems will further improve their applicability and sensitivity for real applications. Manganese complexes are introduced to the electrode surface to enhance the electrochemical detection of DNA. More specifically, when creating a DNA biosensor, the electrode is modified by the electrochemically produced deposition of a manganese complex that is intercalated within the DNA double helix [18].

Our research group has intensively studied the modification of a carbon paste electrode, modified with a manganese complex film (containing thiophene-2-carboxylic acid and triethanolamine) for the electrochemical detection of cyanocobalamin (vitamin B_12_). Using square-wave voltammetry, the test was shown to be selective, cost-effective and sensitive, with no interference from other substances. The method was successfully applied to the analysis of vitamin B_12_ in tablets and food supplements, showing that electrochemical sensors can be used in the future with positive results [19,20,21].

The aim of this study is to explore the effect of loading four different samples of Mn-based NPs on the basis of size, composition and variant organic in halt in order to be applied to the development of a sensitive and reproducible electrochemical dsDNA sensor. Specifically, through a polyol solvothermal process, organic coated Mn-based NPs have been newly prepared and characterized, namely MnCO_3_@OAm (P1), MnCO_3_@TEG(P2) and MnO_2_/Mn_2_O_3_@ TEG (P4), as well as naked MnCO_3_ (P3) NPs (where OAm = oleylamine, TEG = tetraethylene glycol). The physicochemical characterization of Mn-based NPs includes X-ray diffraction (XRD), Fourier transform infrared (FT-IR) and hydrodynamic size and ζ-potential measurements. The freshly prepared Mn-based NPs were initially tested for their interaction with calf thymus DNA (ctDNA) via UV-VIS spectroscopic studies to evaluate their biocompatibility status. The method is based on alterations in the absorption spectrum of ctDNA due to the interaction with the NPs.

Additionally, the novelty of this paper is the use of manganese nanoparticles, fabricated with a simple, cost-effective and green technology and successfully applied in the development of a sensitive and reproducible electrochemical DNA sensor. Therefore, manganese nanoparticles seem to be a promising tool in sensing, since they were successfully applied here. Finally, this work led to the improvement of the properties of pencil graphite electrodes as well as the accurate application of electrochemical techniques.

## 2. Materials and Methods

### 2.1. Chemicals and Reagents for Synthesis of NPs

All the reagents were of analytical grade and were used directly without further purification: potassium permanganate (KMnO_4_) (Aldrich, ≥99.9%, M = 158.03 g/mol), manganese (II) chloride tetrahydrate (MnCl_2_·4H_2_O) (Merck, ≥99.9%, M = 253.15 g/mol)), manganese (II) nitrate hydrate (Mn(NO_3_)_2_·xH_2_O) (Aldrich, ≥98%, M = 178.95 g/mol (anhydrous basis), oleylamine (OAm) (J & K Scientific, Lommel, Belgium, ≥70%, M = 267.5 g/mol), 1,2-propylene glycol (PG) (Merck, ≥99%, M = 76.10 g/mol), tetraethylene glycol (TEG) (Merck, ≥99%, M = 194.23 g/mol) and dimethyl sulfoxide (DMSO) (Aldrich, M = 78.13 g/mol).

### 2.2. Chemicals and Reagents for the Development of DNA Biosensor

Double-stranded deoxyribonucleic acid (dsDNA) and single-stranded deoxyribonucleic acid (ssDNA) from calf thymus were supplied from Sigma-Aldrich (St. Louis, MO, USA) as a lyophilized powder. NaOH, CH_3_COOH, potassium hydrogen phosphate and potassium dihydrogen phosphate were supplied from Merck (Darmstadt, Germany). In this work, ultra-pure water (18 Ω) was used for the preparation of all solutions (Elgastat, High Wycombe, UK), and the chemicals used were of analytical grade. Experiments were performed at room temperature (22.0–25.0 °C).

Buccal swabs obtained from a healthy volunteer were used as the source of DNA. Prior to sampling, the volunteer signed an informed consent form. DNA was extracted from buccal swabs with the use of the QIAampR DNA Mini and Blood Mini Kit (Qiagen, Hilden, Germany). The concentration of DNA was determined by absorption spectrometry, using the extinction coefficients ε260 = 6600 M cm^−1^. 

#### 2.2.1. Solutions

The stock dsDNA and ssDNA solutions were prepared with ultra-pure water (final concentration 1000 mg·L^−1^) and stored at −20 °C until use for DNA biosensor studies. These concentrated DNA solutions were diluted with an acetate buffer (ABS, pH 4.8) to the desired concentrations before the experiments. 

For circular dichroism spectroscopy measurements, a DNA stock solution was prepared by the dilution of DNA with a buffer (containing 150 mM NaCl and 15 mM trisodium citrate at pH 7.0) followed by exhaustive stirring at 4 °C for 3 days and kept at 4 °C for no longer than a week. The stock solution of DNA gave a ratio of UV absorbance at 260 nm of ~1.90 absorbance, indicating that the DNA was sufficiently free of protein contamination. Based on the Beer–Lambert law, the DNA concentration was determined to be approximately 2 × 10^−4^ M.

#### 2.2.2. Apparatus

Most of the electrochemical measurements were performed using an AUTOLAB 12 potentiostat/galvanostat analyzer (Eco Chemie, Utrecht, The Netherlands). The voltammogram obtained from the raw differential pulse data was smoothed by the Savitzky and Golay filter (level 2) of the General Purpose Electrochemical Software (GPES) of Eco Chemie with a moving average baseline correction, using a peak width of 0.01 V. PGE was used as a “working electrode” that contains 3 cm of graphite rod (0.5 mm diameter, Tombo, Japan), and a Rotring model pencil was used as a holder. The three-electrode system includes a pencil graphite electrode (PGE, as a working electrode), an Ag/AgCl electrode (as a reference electrode) and a platinum wire auxiliary electrode obtained from Basi (West Lafayette, IN, USA).

Measurements based on electrochemical impedance spectroscopy were performed with a portable laptop-integrated USB-size mobile potentiostat, GalvanoPlot GX112 (Solar Biotechnology, Izmir, Turkey), after the three-electrode system was established. The GalvanoPlot Suite 1.6.6. software was used in these measurements.

### 2.3. Synthesis of Mn-Based NPs

Sample **(P1)** MnCO_3_@OAm: 0.2 g MnCl_2_·4H_2_O (0.79 mmol) was dissolved in 4 mL of PG and 4 mL of OAm was added. The solution was stirred at 30 °C for 15 min. The resulting solution was transferred into a Teflon-lined stainless-steel autoclave to set out a solvothermal polyol process. The reaction was carried out at 200 °C for 24 h, followed by natural cooling to room temperature. Afterward, the synthetic mixture was centrifuged at 5000× *g* rpm for 20 min and washed three times with disolol, where the supernatants were discarded and a brown precipitate was acquired.

Sample **(P2)** MnCO_3_@TEG and **(P3)** MnCO_3_: 0.2 g KMnO_4_ (1.26 mmol) was dissolved in 8 mL of TEG (for the P2 sample) while 8 mL of PG was used in the case of P3. The mixture (in both cases) was well stirred at 30 °C for 15 min. The supernatant was transferred into a Teflon-lined stainless-steel autoclave to set out a solvothermal polyol process at 200 °C for 24 h.

Sample **(P4)** MnO_2_/Mn_2_O_3_@ TEG: 0.2 g Mn(NO_3_)2·xH_2_O (1.12 mmol) was dissolved in 8 mL of TEG, and mixed well under stirring at 30 °C for 15 min. The resulting solution was transferred into a Teflon-lined stainless-steel autoclave to set out a solvothermal polyol process. The reaction was carried out at 200 °C for 24 h, followed by natural cooling to room temperature.

## 3. Procedure

### 3.1. Characterization of Mn-Based NPs

The crystal structure and crystallite size of synthesized NPs were investigated through X-ray diffraction (XRD) using a Siemens Diffraktometer D5000 performed in the 2*θ* region from 10 to 70°, with monochromatized Cu-Ka X-ray radiation (λ = 1.5418 Å) and a curved crystal graphite monochromator operating at 45 kV and 100 mA; counts were accumulated every 0.02° (2*θ*) with a counting time of 2 s per step. The chemical information of the NPs was determined by Fourier transform infrared spectroscopy (FT-IR) using a Nicolet 6700 FT-IR spectrometer in the wavenumber range of 4000–400 cm^−1^ (2 cm^−1^ resolutions; 30 scans). UV-VIS spectra were recorded with a Jasco V-750 double-beam UV-VIS spectrophotometer. The hydrodynamic size and ζ-potential were measured using a Nano ZS Malvern apparatus at room temperature. The interaction of Mn-based NPs with ctDNA was investigated by circular dichroism spectroscopy employing a Chirascan qCD spectrophotometer in the range of 500–200 nm at room temperature using a 200 μL cuvette with a 0.5 mm path length. The step size, time per data point and the bandwidth/resolution of the device were set at 1 nm, 0.15 s and 1 nm, respectively. CD sample spectra were obtained by subtracting the reference spectrum of the solvent (DMSO) and each spectrum was signal averaged six times.

### 3.2. Interaction with Calf Thymus DNA (DNA)

The DNA stock solution was prepared by dilution of ctDNA with a buffer (containing 150 mM NaCl and 15 mM trisodium citrate at pH 7.0) followed by exhaustive stirring at 4 °C for 3 days and kept at 4 °C for no longer than a week. The stock solution of ctDNA gave a ratio of UV absorbance at 260 nm of ~1.90 absorbance, indicating that the DNA was sufficiently free of protein contamination. Based on the Beer–Lambert law, the ctDNA concentration was determined to be approximately 2 × 10^−4^ M. To explore the interaction between the synthesized Mn-based nanoparticles and ctDNA, UV-vis spectroscopy measurements were performed by keeping the ctDNA concentration constant and incrementally adding a dispersion of NPs (in DMSO) at a concentration of 0.25 g·L^−1^.

### 3.3. Circular Dichroism Spectroscopy Measurements

The circular dichroism spectra (CD) were obtained via a Chirascan qCD spectrophotometer (Applied Photophysics, Surrey, UK) with a 0.5 mm thick cuvette. A stock solution of the sample was prepared at a concentration of 1.0 g·L^−1^ in DMSO. Circular dichroism spectra were first recorded at a constant DNA concentration (1.2 × 10^–3^ M) and then after the addition of increasing percentages of each sample (5–15 μL). Initially, a CD spectrum was corrected for the citrate buffer signal (pH 7.0) and then the results were recorded.

### 3.4. Preparation of Manganese Nanomaterial (Mn-NP)-Modified Disposable Pencil Graphite Electrode(PGE) by Cyclic Voltammetry (CV)

Next, 2000 mg·L^−1^ of stock manganese nanomaterial (Mn-NPs) solutions were prepared with an acetate buffer (ABS, pH: 4.8) and then they were sonicated in a ultrasonic bath for 2 min. The resulting nanomaterials were then diluted to a concentration of 20 mg·L^−1^ with acetate buffer solution (ABS) and deposited onto the PGE surface using cyclic voltammetry (CV) technique with the parameters of 100 mV/s scan rate, 2.5 V step potential, with a potential range from −1.5 V to +1.8 V. Afterwards, the modified electrodes were rinsed twice with ABS.

### 3.5. ssDNA or dsDNA Immobilization at the Surfaces of Bare and Modified PGE

The bare and nanomaterial-modified PGEs were immersed into the plastic vials containing 50 μL of the required concentration of ssDNA or dsDNA prepared in phosphate buffer (PBS) for 15 min. The electrodes were then washed twice with PBS for 2 s to remove unbound DNA from the electrode surface.

### 3.6. Voltammetric Measurements

#### 3.6.1. Cyclic Voltammetry (CV)

The CV measurements were taken for the electrochemical characterization of the electrodes in a 5 mM potassium ferricyanide [Fe(CN)_6_]^3−/4−^ solution with the parameters of 50 mV/s scan rate, with a potential range from −0.25 V to +0.65 V.

#### 3.6.2. Differential Pulse Voltammetry (DPV)

The oxidation signal of the guanine in the DNA used in the experiments was obtained by the DPV technique at approximately +1.0 V. Measurements were carried out in the potential range of +0.75 V to +1.25 V in an acetate buffer (ABS), with a pulse amplitude of 50 mV and a scan rate of 16 mV/s.

#### 3.6.3. Electrochemical Impedance Spectroscopy (EIS)

Impedance measurements from EIS were obtained in 5 mM [Fe(CN)_6_]^3−/4−^ including 0.1 M KCl. The frequency range was applied between 50,000 Hz and 0.1 Hz during the impedance measurement at a potential of +0.23 V, with an Eac of 30 mV (amplitude) and 60 logarithmically measured points.

## 4. Results and Discussion

### 4.1. Synthetic Aspects and Characterization

**Figure 1:**
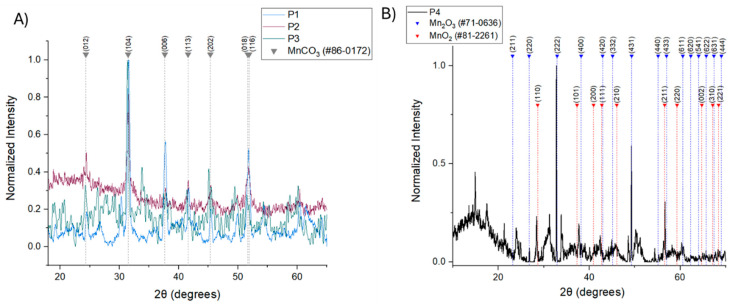
X-ray diffraction graphs of synthesized Mn-based NPs (P1, P2, P3, P4). (**A**) X-ray diffraction graphs for P1, P2, P3 and MnCO_3_ (**B**) X-ray diffraction graphs for P4, Mn_2_O_3_, MnO_2_.

The polyol process through a solvothermal procedure appeared to be a versatile route for the preparation of metal-based NPs [22]. In the present study, depending on the choice of the metal precursor, MnCl_2_·4H2O or KMnO_4_, and on the different molecular weights of polylols, such as PG and TEG, respectively, the redox system reacted extensively up to the point that the oxygen was replaced by carbon dioxide in the reaction vessel in the autoclave. As a result, the presence of the MnCl_2_·4H_2_O precursor and PG (Figure 1A) resulted in the formation of hexagonal MnCO_3_ NPs with space group R-3c, as shown by the diffraction peaks that correspond to the (012), (104), (006), (110), (113), (202), (024), (018) and (116) planes of MnCO_3_ rhodochrosite phase (JCPDS no. 86-0172)^1^. Lattice parameters were estimated at α = b = 4.773 Å, c = 15.642 Å, α = β = 90° and γ = 120° [23,24]. In the case of the KMnO_4_ precursor, both polyols employed also resulted in the synthesis of hexagonal MnCO_3_ NPs as shown by the diffraction peaks that correspond to the (012), (104), (006), (113), (202), (018) and (116) planes of the MnCO_3_ rhodochrosite phase (JCPDS no. 86-0172). Lattice parameters were estimated at α = b = 4.773 Å, c = 15.642 Å, α = β = 90° and γ = 120°. The absence of any additional peaks in the diffraction pattern serves as confirmation of the high purity of the manganese carbonate (MnCO_3_) crystallite.

On the other hand, when Mn(NO_3_)_2_·xH_2_O in the presence of TEG (Figure 1B) led to the synthesis of cubic-phase bixbyite with space group Ia-3, Mn_2_O_3_ NPs (92%) with traces of cubic phase pyrolusite, MnO_2_ (8%), was detected, as indicated by the diffraction peaks that correspond to the (211), (220), (222), (400), (420), (332), (431), (440), (433), (611), (620), (541), (622), (631) and (444) planes of the Mn_2_O_3_ bixbyite phase (JCPDS no. 71-0636) and the (110), (101), (200), (111), (210), (211), (220), (002), (310) and (221) planes of the MnO_2_ pyrolusite phase (JCPDS no. 81-2261). Lattice parameters were estimated at α = b = c = 9.4146 Å, α = β = γ = 90° for Mn_2_O_3_ [25], and α = b = 4.4041 Å, c = 2.8765 Å, α = β = γ = 90° for MnO_2_ [26]. Additionally, the diffraction peaks that are observed below 20 degrees indicate the crystallization of organic compounds (tetraethylene glycol and/or oxidation derivatives) on the NPs’ surface [27].

The crystallite size of all phases was calculated from the Scherrer equation, D = 0.891λ/β cosθ, and the full width at half-maximum of the main peak of each crystallite phase. For P1 the crystallite size is 48 nm, for P2 it is 26 nm, for P3 it is 25 nm and for P4 it is 140 nm, respectively.

**Figure 2:**
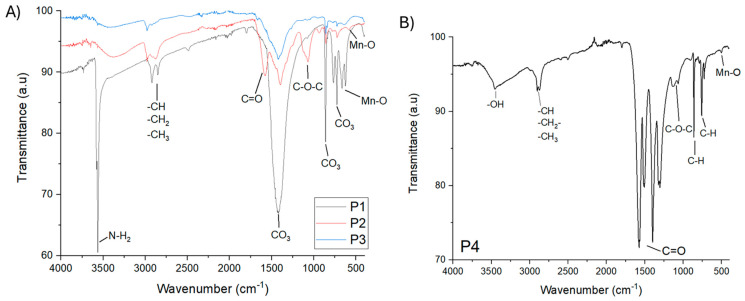
IR spectra of synthesized Mn-based NPs (P1, P2, P3 and P4). (**A**) Ir spectra for P1, P2, P3 (**B**) IR spectra for P4.

The presence of the organic coated Mn-based NPs as well as the characteristic peaks of -CO_3_ groups were certified by FTIR spectra (Figure 2). Common features in P1-P3 (Figure 2A) are the peaks at 1418, 861 and 727 cm^−1^ that are characteristic of the CO_3_ groups [28] and the weak absorptions at about 660 and 620 cm^−1^ attributed to the lattice vibrations of Mn–O bond. Additionally, at **P1** (Figure 2A), the strong absorptions at 3566 cm^−1^ and 3562 cm^−1^ are attributed to the N-H2 bond, which confirms the existence of OAm on the surface of the NPs. Also, the absorptions at 2923 and 2853 cm^−1^ are attributed to the bending vibrations of the methylene groups -CH_2_- and -CH_3,_ which are derived from OAm [29,30]. In the spectrum of the **P2**, the absorptions at 2981 and 2876 cm^−1^ are attributed to the bending vibrations of methylene groups, derived from propylene glycol. The absorbance at 1580 cm^−1^ is attributed to the C=O bond stretching vibration and the absorbance at 1072 cm^−1^ is attributed to the C-O-C bond stretching vibration originating from the oxidation products of tetraethylene glycol, which is oxidized to aldehyde and ketone derivatives and oxalic acid derivatives [31]. The naked nature of **P3** is evidenced.

The **P4** spectrum (Figure 2B) is very different. The broad band at 3460 cm^−1^ is allotted to the presence of hydroxyl groups. The peaks at 1118 and 1062 cm^−1^ correspond to the C-O-C vibration and the absorptions at 861 and 759 cm^−1^ are attributed to the in-plane C-H bond-bending vibration, which are derived from TEG oxidation derivatives. The peaks at 1575, 1512 cm^−1^ and at 1397, 1301 cm^−1^ suggest the presence of the oxidation derivatives of tetraethylene glycol. The polyol oxidation mechanism of TEG in the preparation of inorganic-based nanostructures has been previously explored by us [32]. Generally, the moving force of the polyol process is the redox system that is setting up amongst the polyols and the metal ions and specifically the oxidation of the polyols and the reduction in metal precursors. The mechanism follows the formation of polyol–metal complexes that decompose, giving rise to the nucleation and growth of NPs. Both the formation and decomposition of these intermediate polyol–metal complexes are very sensitive to synthetic regulations. Thus, different oxidized derivatives/fragments like glycolaldehyde, glycoxylic acid, glycolic acid, oxalic acid and oxalates are presented based on the specific synthetic conditions [33,34]. The absorption at 504 cm^−1^ is attributed to the vibrations of the Mn–O bond.

**Figure 3:**
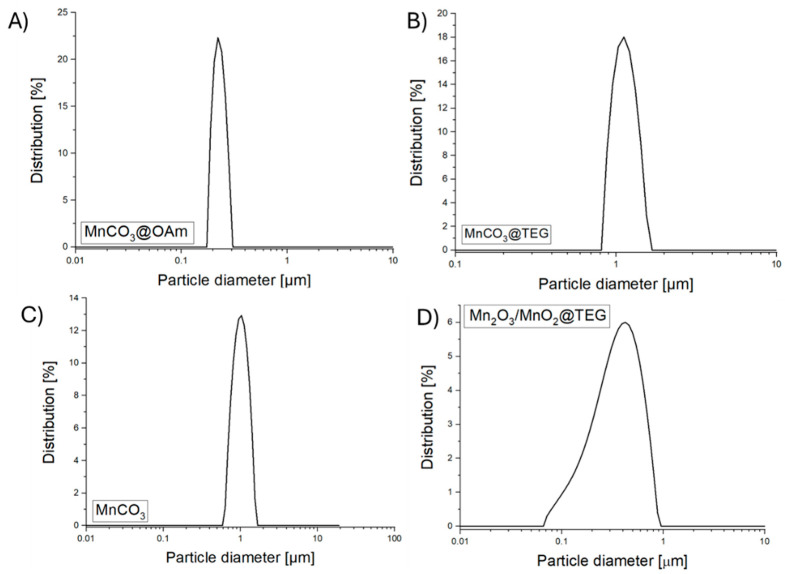
Size distribution of Mn-based NPs (**A**) MnCO_3_@OAm, (**B**) MnCO_3_@TEG, (**C**) MnCO_3_ and (**D**) MnO_2_/Mn_2_O_3_@TEG in DMSO.

The colloidal stabilization of the synthesized Mn-based NPs in DMSO was confirmed by measuring their average hydrodynamic particle size (Mean ± PDI) and ζ-potential (Figure 3). The average size of MnCO_3_@OAm (P1) NPs found 293 ± 0.1 nm, while the ζ-potential was determined to be slightly positive +8.7 ± 1 mV. In contrast, MnCO_3_@TEG (P2) NPs display a larger average size of 1.31 ± 0.123 μm, accompanied by an average ζ-potential of +2.5 ± 0.9 mV. The increased hydrodynamic size can be attributed to particle aggregation. MnCO_3_ (P3) NPs have an average size of 920 ± 0.213 nm, with an average value of ζ-potential +9.7 ± 1.1 mV, similarly indicating aggregation contributing to the larger hydrodynamic size. Finally, MnO_2_/Mn_2_O_3_@TEG (P4) NPs possess an average size of 322 ± 0.29 nm with a slightly negative value of ζ-potential −4.6 ± 1.3 mV.

### 4.2. Interaction of Mn-Based NPs with DNA by UV-VIS

The chromophore groups present on the bases of DNA lead to a UV-VIS absorption spectrum with a maximum absorption of about 260 nm of DNA. The binding interaction of DNA with nanoparticles or small ligands is governed by two main phenomena in the DNA spectrum, known as hyperchromatic (increase in absorbance) and hypochromatic (decrease in absorbance) phenomena. The hyperchromatic effect indicates the unwinding/partial unwinding of the DNA secondary structure upon non-covalent interaction with the nanoparticles. This phenomenon occurs due to the binding of the grooves or the electrostatic interaction of the negatively charged DNA with the positively charged nanoparticles. On the other hand, the hypochromic effect is attributed to the stabilization of the helical structure of the DNA through electrostatic or intercalated interaction of the small particles. Furthermore, bathochromism (red shift) is an indication of stabilization of the DNA structure, whereas hypsochromism (blue shift) implies structural destabilization. The hypochromic effect along with the red shift is mainly due to the interference of the small particles with the DNA helix [35,36].

**Figure 4:**
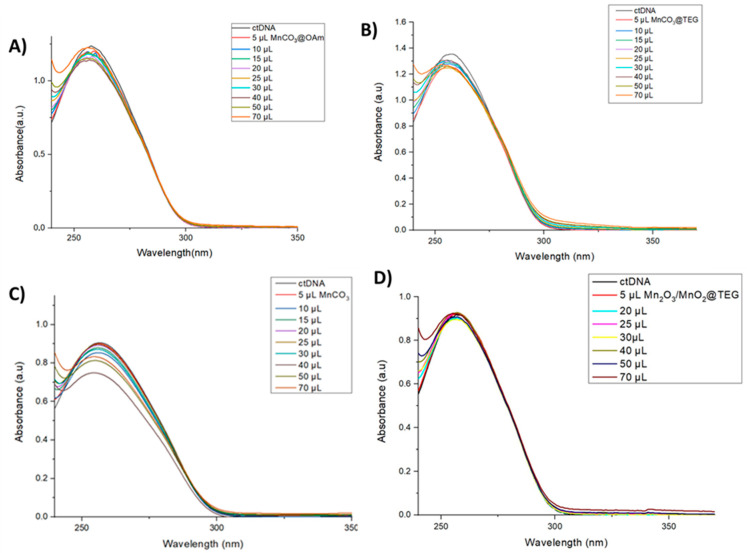
UV–vis spectra of ctDNA solution (1 × 10^−4^ M) in the presence of increasing amounts of DMSO dispersion of Mn-based NPs (0.25 g·L^−1^). The arrows show the changes upon increasing amounts of NPs dispersion. (**A**) MnCO_3_@OAm, (**B**) MnCO_3_@TEG, (**C**) MnCO_3_ and (**D**) MnO_2_/Mn_2_O_3_@TEG.

Upon the addition of the MnCO_3_ NPs dispersion (Figure 4A–C), the absorption bands display hypochromism and a slight red shift, suggesting electrostatic interactions or interference between the nanoparticles and DNA. Notably, MnCO_3_ NPs (P3) show the greatest degree of hypochromism and the most pronounced red shift, followed by MnCO_3_@OAm NPs (P1), with MnCO_3_@TEG NPs (P2) exhibiting the least effect. Given their size and positive zeta potential, it is likely that these interactions are electrostatic in nature, as DNA carries a negative charge.

On the other hand, based on the UV-vis spectra of the MnO_2_/Mn_2_O_3_@TEG NPs (Figure 4D), no significant shifts in the absorption maxima were observed across all experimental conditions, indicating no detectable interaction between the NPs and DNA.

The DNA interaction of MnCO_3_ NPs was further investigated employing circular dichroism spectroscopy. The positive band at 275 nm and the negative band at 245 nm in the CD spectrum are due to base stacking (p-p) and the clockwise helical conformation of B-DNA, respectively [37,38]. Interference enhances the intensities of both bands, whereas simple groove binding and the electrostatic interaction of the NPs with DNA exhibit less or no changes in the bands due to base stacking and helical conformation of B-DNA [39].

**Figure 5:**
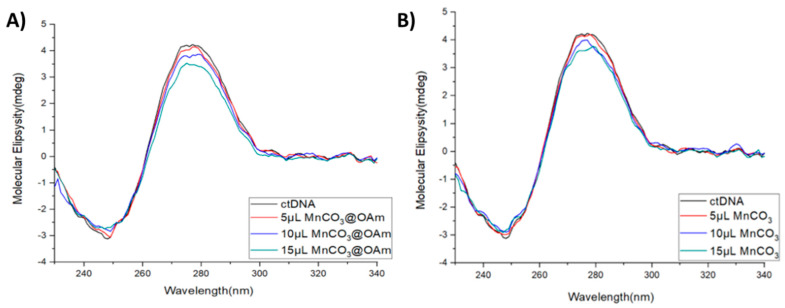
CD spectrum of ctDNA (1.2 × 10^−3^ M) in the absence (black line) and presence of (**A**) MnCO_3_@OAm and (**B**) MnCO_3_ NPs.

Upon the addition of MnCO_3_@OAm (P1) and pure MnCO_3_ NPs (P3) dispersion to the ctDNA solution, a reduction in the intensity of both absorption bands is observed (Figure 5A,B). The effect is more pronounced for MnCO_3_@OAm NPs followed by the pure MnCO_3_ NPs. These findings indicate that MnCO_3_ NPs may interact with ctDNA, potentially through groove binding or electrostatic interactions [40].

### 4.3. Electrochemical Characterization of the Mn-Based Nanomaterials

Since Paleček discovered the electroactivity of nucleic acids (NAs), an extensive investigation of the electrochemistry of nucleic acids (NAs) began. This research led to a series of voltammetric approaches, both for the rapid and inexpensive quantification of DNA and for the detection of changes in its structure. Thus, electrochemical biosensors have attracted enormous attention as they combine the analytical features of electrochemical methods with the ability to recognize DNA sequences. The application of electrochemical techniques offer enhanced selectivity and sensitivity compared to optical techniques.

In this study conducted with different types of manganese nanomaterial-modified PGE electrodes, an evaluation was made according to the increased ferricyanide or guanine oxidation signals obtained compared to the bare electrode.

The electrochemical characterization of the bare pencil graphite electrode and the manganese nanoparticle-modified PGE surfaces were carried out using the CV and EIS techniques and then compared to bare PGE [41].

**Figure 6:**
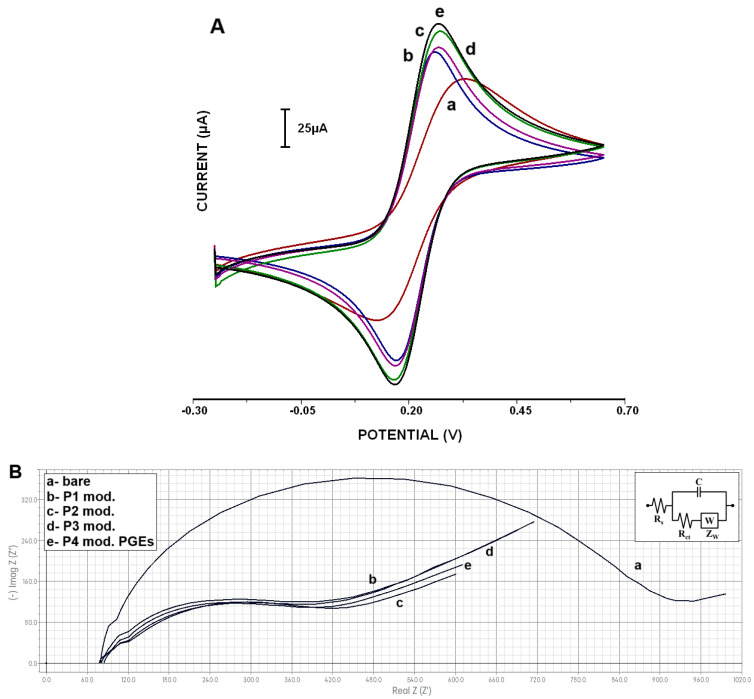
(**A**). Cyclic voltammograms representing the average anodic peak current (Ipa) at a scan rate of 50 mV/s obtained from (a) bare PGE and 20 mg·L^−1^ Mn-NP-modified PGEs, and (**B**). Nyquist plots for (a) bare PGE and Mn-NP-modified PGEs as (b) P1-PGE, (c) P2-PGE, (d) P3-PGE, (e) P4-PGE in a 5 mM [Fe(CN)_6_]^3−/4−^ solution.

The average Ia of bare PGE was found to be 76 ± 3.30 μA with the relative standard deviation (RSD %) as 4.36% (Figure 6A-a). On the other hand, the average anodic currents were obtained as 106.33 ± 2.49 µA (RSD; 2.35%) and 118.28 ± 3.09 (RSD; 2.61%), 107.98 ± 3.59 µA (RSD; 3.32%) and 121.85 ± 3.12 µA (RSD; 2.56%) after the modification of P1 (Figure 6A-b), P2 (Figure 6A-c), P3 (Figure 6A-d) and P4 (Figure 6A-e) types of Mn-NPs on the PGE surfaces, respectively (n = 4).

The highest enhancement of the signal of [Fe(CN)_6_] ^3−/4−^ in the I_a_ value was observed after applying the 20 mg·L^−1^ P4-type Mn-NP (Mn_2_O_3_/MnO_2_@TEG) modification to the PGE surface (121.85 ± 3.12 µA (RSD; 2.13%; Figure 6A-e) compared to the other types of nanomaterial-modified PGEs. These results, in which the electrochemical signals increased significantly, indicate that Mn-NPs increased the effective electroactive surface area (ESA), see Table 1.

Figure 6B represents the electrochemical impedance spectra of bare PGE and Mn-NP-modified PGEs, and the inset shows the equivalent circuit model of the related data. In the Nyquist plot of EIS parameters are as follows: Rct was the charge transfer resistance at the interface where the reaction takes place, Rs is the resistance of the solution, C is the constant phase element related to the double-layer charge capacitance, W(Z_w_) is the diffusion of the ferri/ferrocyanide from the solution to the electrode surface. In the evaluation of the Nyquist plot, the diameter of the semicircle of the charge transfer resistance (R_ct_) resulting from the reaction was used.

Similarly to the CV measurements, significant signal differences were obtained between bare and Mn-NP-modified electrodes in the EIS data. After the modification of clear Mn-NPs, diminished R_ct_ values (Figure 6B-b–e) were observed compared to the bare electrode. After the modification of the PGE surface with P1 (MnCO_3_@OAm)-, P2 (MnCO_3_@TEG)-, P3 (MnCO_3_)- or P4 (MnO_2_/Mn_2_O_3_@TEG)-type Mn-NPs, it was found that the Rct value decreased by almost 50% for each modification. When these nanomaterial modifications were compared with each other, it was observed that electrodes modified with P4 and P2 gave slightly smaller R_ct_ values than those modified with P1 and P3. These results obtained with EIS support those obtained with CV (Figure 6A). Electrochemical impedance graphs with adjustments made by the equivalent circuit for impedance measurements made with the Galvanoplot USB-type potentiostat device have also been added to the supporting information section (Figure S1). The significant signal difference obtained between bare and nanomaterial-modified electrodes can be explained by the high surface area provided by the relevant modification and the greater electron transfer and improved conductivity in the double-layer region of Mn-NPs.

### 4.4. The Effect of the Scan Rate on the Ferricyanide Signal and the Calculation of the Electroactive Surface Area (ESA)

Mn-NP-modified PGE surface properties were also investigated by CV using a K_4_[Fe(CN)_6_]/K_3_[Fe(CN)_6_] solution. According to the results obtained from CV measurements, when compared to the bare electrode, nearly 1.4, 1.6, 1.43 and 1.6 times higher peak currents were observed as P1-, P2-, P3- and P4-modified PGE were used, respectively (Figure S2). In addition, as can be clearly seen from the figures, ΔEp_[a-c]_ values decrease with the modification of Mn-NPs, and more well-defined peaks are obtained compared to the unmodified electrode. This change in the anodic and cathodic peak points also proves that the surface properties of the nanomaterial-modified electrodes have changed [42]. For surface morphologies of unmodified and Mn-NP-modified pencil graphite electrodes, see the scanning electrochemical microscopy (SEM) measurements in Figure S3.

The effective electroactive surface areas (ESAs) of bare and Mn-NP-modified PGEs were calculated according to the Randles–Sevcik equation [43] and presented in Table 1. The results showed that the manganese nanoparticle-modified PGE surfaces are more advantageous than unmodified ones. According to the equation, the ESA of the unmodified electrode surface was found as 0.092 cm^2^, while the ESA of PGE modified with P4-type Mn-NPs (MnO_2_/Mn_2_O_3_@TEG) with the largest surface area was calculated as 0.147 cm^2^.

Ip = (2.69 × 10^5^) · n^3/2^ · A · C · D^1/2^ · υ^1/2^ (1)

Additionally, the anodic peak potential (Epa) values for the bare and Mn-NP-modified electrodes were as follows: 0.316 V for bare, 0.258 V for P1-, 0.270 V for P2-, 0.265 V for P3-, 0.263 V for P4-modified electrodes. The amount of leftward shift in the Epa value was measured as 58, 46, 51 and 53 mV after the P1-, P2-, P3- and P4-type nanoparticle modification on the electrode surface. According to the results, a shift to the left is observed in the Epa after manganese nanoparticle modification compared to bare PGE, indicating that Mn-NPs have a facilitating effect on Fe^2+^ oxidation. This also suggests that Mn-NPs increase the electron transfer rate between the Nernst diffusion layer and the solution interface.

### 4.5. Biosensor Applications

#### 4.5.1. Electrochemical Detection of dsDNA Using Mn-NP-Modified PGE

The dsDNA (ctDNA) detection performances of PGEs modified with different types of Mn-NPs were compared using the DPV technique according to the guanine signal magnitudes obtained after dsDNA immobilization on these surfaces [44].

**Figure 7:**
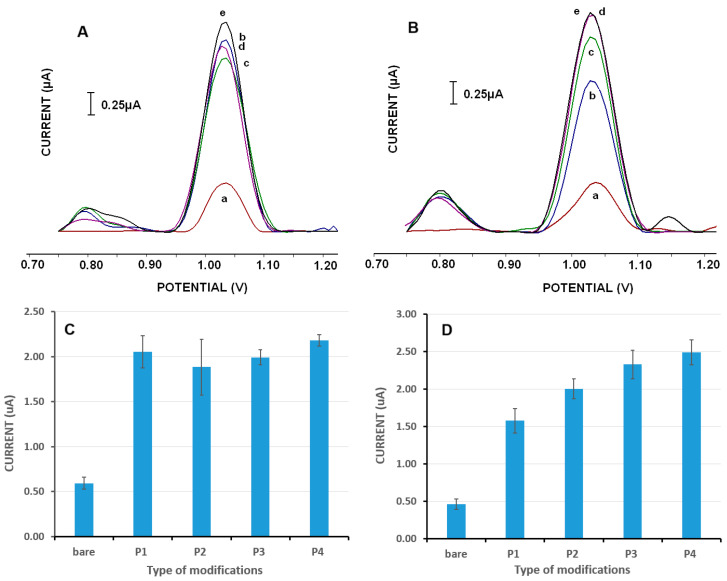
Differential pulse voltammograms (**A**,**B**) and histograms (**C**,**D**) representing the guanine oxidation signals obtained from (a) bare PGE, (b) P1 (MnCO3@OAm)-modified PGE, (c) P2 (MnCO_3_@TEG)-modified PGE, (d) P3 (MnCO_3_)-modified PGE and (e) P4 (MnO_2_/Mn_2_O_3_@TEG)-modified PGE in the presence of 50 mg·L^−1^ (**A**,**C**) or 100 mg·L^−1^ (**B**,**D**) dsDNA on the electrode surfaces. Measurements were carried out in the potential range of +0.75 V to +1.25 V in an acetate buffer, with a pulse amplitude of 50 mV and a scan rate of 16 mV/s.

The changes in the guanine signals were obtained at about 1.0 V from different types of Mn-NP-modified PGEs at 50 mg·L^−1^ dsDNA concentration for 15 min immobilization time (Figure 7A,C). The average signals were obtained as 2.05 ± 0.18 μA (% RSD (n = 6); 8.72%); 1.88 ± 0.31 μA (% RSD (n = 8); 16.4%); 1.99 ± 0.08 μA (% RSD (n = 6); 4.11%); 2.18 ± 0.11 μA (% RSD (n = 4); 4.91%); and 0.59 ± 0.07 μA (% RSD (n = 5); 11.27%) after the immobilization of dsDNA onto the surface of P1 (MnCO_3_@OAm)-, P2 (MnCO_3_@TEG)-, P3 (MnCO_3_)- or P4 (MnO_2_/Mn_2_O_3_@TEG)-modified and bare PGE, respectively. A significant increase was observed after DNA immobilization on Mn-NP-modified PGE (Figure 7A; b–e).

According to Figure 7A, increments were monitored in the voltammetric signals obtained from the Mn-NP-modified PGE surfaces; these increases were approximately 3.5 times higher for the P1-modified PGE, 3.2 times higher for the P2-modified PGE, 3.4 times higher for the P3-modified PGE and 3.6 times higher for the P4-modified PGE compared to the responses in the nanomaterial-free PGE.

When the guanine signals obtained from 100 mg·L^−1^ dsDNA immobilized electrode surfaces are evaluated (Figure 7B,D), the average signals were obtained as 1.58 ± 0.16 μA (% RSD; 10%); 2.00 ± 0.13 μA (% RSD; 6.5%); 2.33 ± 0.19 μA (% RSD; 8%); 2.49 ± 0.17 μA (% RSD; 6.6%); and 0.46 ± 0.07 μA (% RSD; 15%) after the immobilization of dsDNA onto the surface of P1-, P2-, P3-, and P4-modified and bare PGE, respectively (n = 3). A significant increase was observed after DNA immobilization on Mn-NP-modified PGE (Figure 7B, b–e).

The increases in electrochemical signals obtained from Mn-NP-modified PGE surfaces were approximately 3.4-fold higher for P1-modified PGE, 4.3-fold for P2-modified PGE, 5.1-fold for P3-modified PGE and 5.4-fold for P4-modified PGE compared to the responses in PGE without nanomaterials, respectively.

The increases in dsDNA immobilization on electrode surfaces containing Mn-NPs compared to bare PGE clearly show that the modification of Mn-NPs enlarges/increases the surface area. Another point is that nanomaterials can also adsorb biomolecules electrostatically at a high rate because they have high surface free energy and large surface area. With these features, they can play an important role in biomolecule immobilization for new-generation biosensor designs. Therefore, in this study, it is thought that the immobilization of negatively charged dsDNA on Mn nanomaterials with possible positive charges occurs by electrostatic interaction-based adsorption. These results are in agreement with results obtained in UV-vis and CD interaction studies.

When these results are evaluated, it is shown that the P4-type nanomaterial-modified PGE surface provides more sensitive dsDNA detection than the other types of Mn-NP-modified PGE surfaces. However, it is also seen that all Mn-NP-modified surfaces have high DNA-binding abilities and provide quite sensitive determination compared to the bare electrode.

#### 4.5.2. Detection Sensitivity for Double-Stranded DNA (dsDNA)

**Figure 8:**
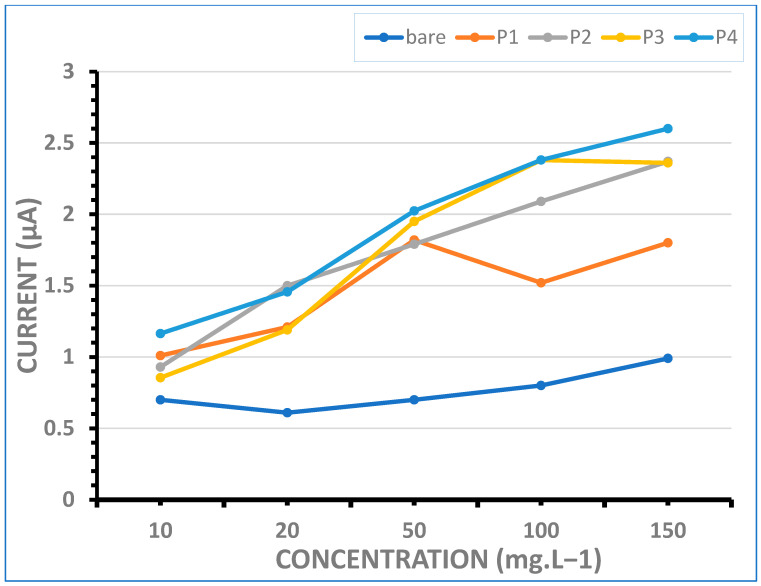
Line graphs presenting the average guanine oxidation signals obtained at about +1.0 V by using DPV in the conditions of increasing concentration of dsDNA immobilization between 10 and 150 mg·L^−1^; the bare PGE (light blue line), P1-modified (orange line), P2-modified (gray line), P3-modified (yellow line) and P4-modified PGEs (blue line). Other conditions are as in Figure 7.

The effect of dsDNA concentration upon guanine oxidation responses obtained from bare and Mn-NP-modified PGEs was studied. The line graphs showing the magnitude of guanine peaks after the immobilization of the biomolecule on the bare and Mn-NP-modified electrodes in the concentration range of 10–150 mg·L^−1^ are presented in Figure 8. A steady increase in the guanine oxidation signal was observed on almost all of the Mn-NP-modified electrode surfaces up to 100 mg·L^−1^ dsDNA immobilization (except P1), and after this concentration, there was a lower signal increase observed in the response (except P3) due to the steric hindrance effect of high concentrations (Figure 4, lines P1, P2, P3 and P4).

At low DNA concentrations, such as 20 mg·L^−1^, the guanine oxidation responses obtained from P2- and P4-modified electrode surfaces were higher (Figure 8; the gray and blue lines; 1.50 μA and 1.46 μA, respectively) than those from P1- and P3-modified ones (Figure 8; the orange and yellow lines; 1.21 μA and 1.19 μA, respectively). Therefore, when dsDNA analysis at lower concentrations is required, it would be appropriate to use electrodes containing P2 and P4.

When the results obtained for 50 mg·L^−1^ dsDNA concentration are examined, responses were obtained as 1.82, 1.79, 1.95 and 2.02 microamperes for P1, P2, P3 and P4, respectively. The highest guanine oxidation value was obtained with P4-modified PGE as approximately 2.02 μA.

On the other hand, at 100 mg·L^−1^ and higher dsDNA concentrations, the highest current response was obtained from the P3- or P4-modified electrode. Furthermore, the responses obtained at 100 mg·L^−1^ are the same with the P3- or P4-modified electrodes (2.38 μA), and there is a slight difference between these two responses with the P2-modified one (2.09 μA). However, when the results obtained with 150 mg·L^−1^ ds DNA are evaluated, it is seen that the highest response is obtained from the P4-modified electrode as 2.6 μA. The responses obtained with the P2- and P3-modified electrodes for 150 mg·L^−1^ ds DNA are also relatively high (2.37 μA and 2.36 μA). This is evidence that the surface area of the P4- and also P2- and P3-modified electrodes have been significantly expanded due to the effect of nanomaterial modification and have become suitable for a high amount of DNA binding. However, when analysis at higher concentrations is required, it would be appropriate to use electrodes containing P4-type Mn-NPs (MnO_2_/Mn_2_O_3_@TEG).

Another point is that, regardless of dsDNA concentration, Mn-NP-modified electrode surfaces have been found to provide high signal acquisition due to DNA binding up to 2.4 to 3 times more than the bare electrode. The greatest difference in the responses obtained with bare and modified electrodes was at 100 mg·L^−1^ and was observed as nearly 3 times between P3- and P4-modified and bare PGEs, 2.6 times between P2-modified and bare PGEs and 1.9 times between P1-modified and bare PGEs, respectively. The optimum concentration for dsDNA detection to the P3- or P4-modified PGE biosensor was found as 100 mg·L^−1^ with a good reproducibility of 7% and 9.5% of RSD, n = 3, respectively.

**Figure 9:**
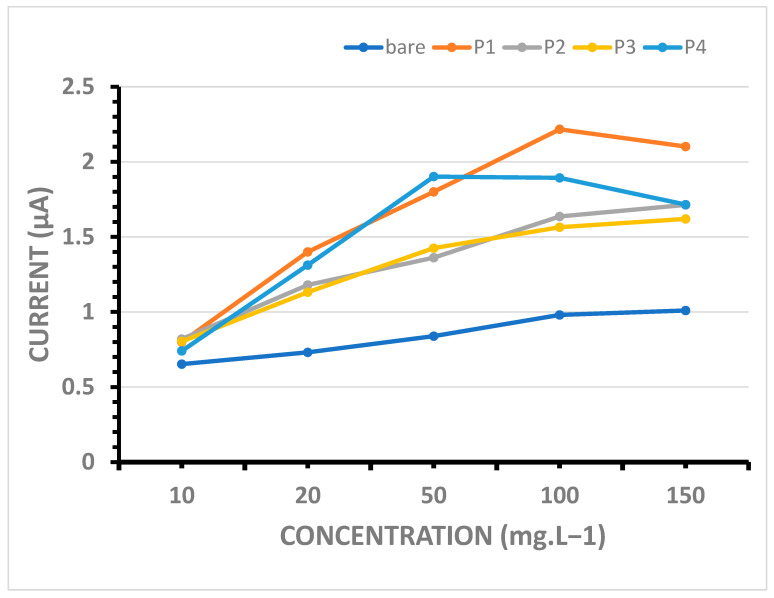
Line plots showing the average guanine oxidation responses observed by using DPV in the conditions of increasing concentrations of ssDNA immobilization between 10 and 150 mg·L^−1^; the bare PGE (light blue line), P1-modified (orange line), P2-modified (gray line), P3-modified (yellow line) and P4-modified PGEs (blue line). Other conditions as in Figure 7.

According to the *LOD* definition of IUPAC and the literature [45,46] using the linear part of the calibration plots obtained from Figure 8 (P4 line, MnO_2_/Mn_2_O_3_@TEG) and Figure 9 (P1 line, MnCO_3_@OAm), the detection limit (*LOD*) of the P4-modified biosensor was calculated as 7.86 μg·L^−1^ for dsDNA and the *LOD* of the P1-modified sensor was calculated as 3.49 μg·L^−1^ for ssDNA based on Equation (2):

*LOD* = 3*Sb*/*q* (2)

In the equation, *Sb* shows the standard deviation (SD) of the blank (for this study, SD of the buffer response in the absence of DNA was used) and *q* represents the slope of the calibration graph.

#### 4.5.3. Detection Sensitivity for Single-Stranded DNA (ssDNA)

**Figure 10:**
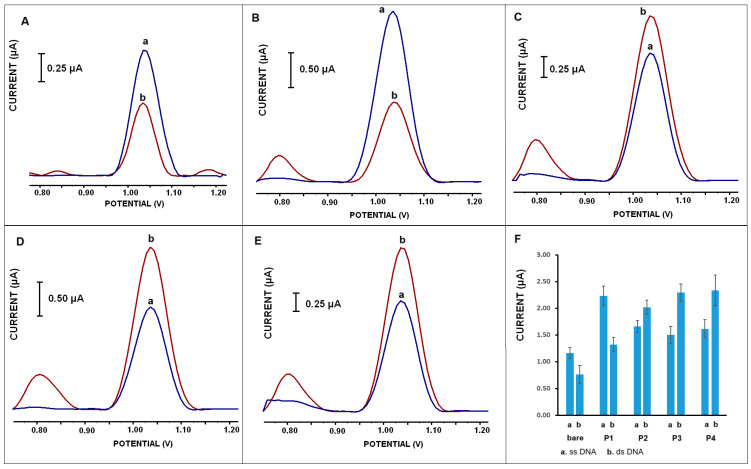
The guanine responses obtained from bare and nanomaterial-modified PGE surfaces as (**A**) shows bare, (**B**) shows P1-modified, (**C**) shows P2-modified, (**D**) shows P3-modified and (**E**) shows P4-modified ones after ss DNA immobilization (a’s of Figure 10) and after dsDNA immobilization (b’s of Figure 10). (**F**) shows the histogram of all results. Other conditions are explained in Figure 7.

The effect of ssDNA concentration on the magnitude of the guanine peaks obtained from unmodified and the Mn-NP-modified biosensor was investigated. The line graphs voltammograms presenting the magnitude of guanine oxidation signals in the presence of the ssDNA on the bare or Mn-NP-modified PGEs in the concentration range of 10–150 mg·L^−1^ are shown in Figure 5. Similarly to the experiment conducted with dsDNA in Figure 3, a gradual increase in the guanine response was obtained on nearly all Mn-NP-modified PGE surfaces (except P4) up to 100 mg·L^−1^ ssDNA immobilization, and after this value, while some decrease was observed in the P4 and P1 signals, a slight increase was observed in the signals obtained from the P2- and P3-modified surfaces due to the steric hindrance caused by the high analyte concentration on the PGE surface (Figure 10, 150 mg·L^−1^ ssDNA, P1, P2, P3 and P4 lines).

At low DNA concentrations, such as 20 mg·L^−1^, the guanine oxidation responses obtained from P1- and P4-modified electrode surfaces were higher (Figure 9; the orange and blue lines; 1.40 μA and 1.31 μA, respectively) than those from P2- and P3-modified ones (Figure 10; the gray and yellow lines; 1.18 μA and 1.13 μA, respectively). Therefore, when analysis at lower concentrations is required, it would be appropriate to use electrodes containing P1 and P4.

When the results obtained for 50 mg·L^−1^ ssDNA concentration are examined, responses obtained from P1- and P4-modified electrode surfaces were still higher (Figure 9; the orange and blue lines; 1.80 μA and 1.90 μA, respectively) than those from P2- and P3-modified ones (Figure 9; the gray and yellow lines; 1.36 μA and 1.43 μA, respectively). However, at 100 mg·L^−1^ ssDNA concentration, the highest current response was obtained from the P1-modified electrode up to 2.22 μA. This shows that ss DNA has an affinity for the P1-modified electrode and binds to its surface more than other types of Mn-NP-modified PGEs. On the other hand, when the results obtained with 150 mg·L^−1^ ss DNA are evaluated, it is seen that the highest response is still observed with the P1-modified electrode as 2.1 μA; this situation can be explained by the fact that P1 is the only donor of the -NH_2_ group as it is coated with oleylamine. However, the obtained results showed that the affinity of ssDNA to the P1-modified surface containing oleylamine is much higher than the surfaces modified with other types of NPs used in the study. Responses obtained with the other three types of nanomaterial-modified electrodes are also relatively good.

As a result of this experiment, it was observed that the highest difference between the responses obtained with bare and modified electrodes at 100 mg·L^−1^ ssDNA concentration was approximately 2.3-fold between P1-modified (MnCO_3_@OAm) and bare PGEs with a good reproducibility 8% of RSD, n = 3).

#### 4.5.4. Investigation of the Affinities of Nanomaterial-Modified Surfaces to ssDNA or dsDNA

The changes in DPV peak heights of guanine obtained with the single-stranded DNA (a’s of Figure 10)- or double-stranded DNA (b’s of Figure 10)-modified Mn-NPs covered PGEs are presented in Figure 10.

The guanine oxidation peaks obtained with the ssDNA-modified PGE (a’s of Figure 10A,B) were higher than the ones observed with the dsDNA-modified electrode (b’s of Figure 10A,B). The peak current obtained for them in dsDNA-modified PGE decreased by approximately 33.6% (Figure 10A for bare PGE) and 53.3% (Figure 10B for P1-mod. PGE), respectively. On the other hand, the magnitude of guanine signals obtained with the dsDNA-immobilized PGE (b’s of Figure 10C–E) was higher than the ones observed with the ssDNA-containing electrode (a’s of Figure 10C–E). Here, the peak heights obtained with PGE immobilized with ssDNA decreased by approximately 18% (Figure 10C for P2-mod. PGE), 35% (Figure 10D for P3-mod. PGE) and 35% (Figure 10E for P4-mod. PGE), respectively, when compared to dsDNA. When all findings are evaluated, it can be explained that single-stranded or double-stranded DNA exhibits different affinity and binds to electrode surfaces modified with Mn-NPs of different properties. In this direction, it was determined that ssDNA exhibited a higher affinity to the surface containing P1 compared to those modified with other Mn-NPs (P2, P3 and P4). In Figure 10F, the ss/dsDNA affinities for the modified electrodes with the Mn-NP types used in the study are shown collectively and presented as comparable to each other. Accordingly, while the affinity of ssDNA to the P1-modified surface is higher, the affinity and binding of dsDNA to the P3- or P4-modified electrode is higher than P1. Although dsDNA shows slightly higher affinity than ssDNA to the P2-modified surface, it can be evaluated that there is equal affinity for both ss and dsDNA (Figure 10F).

#### 4.5.5. dsDNA Detection at Solution Phase

In this group of experiments performed in the solution phase, two different solutions were prepared for measurements such as a buffer (for blank measurement) and a buffer containing dsDNA (for guanine signal measurement). PGE tips modified with Mn-based nanomaterials were interacted with these solutions in preparation before the experiment for 1 min, and CV measurements were taken in the same solution to determine the dsDNAs bound to the electrode surface.

**Figure 11:**
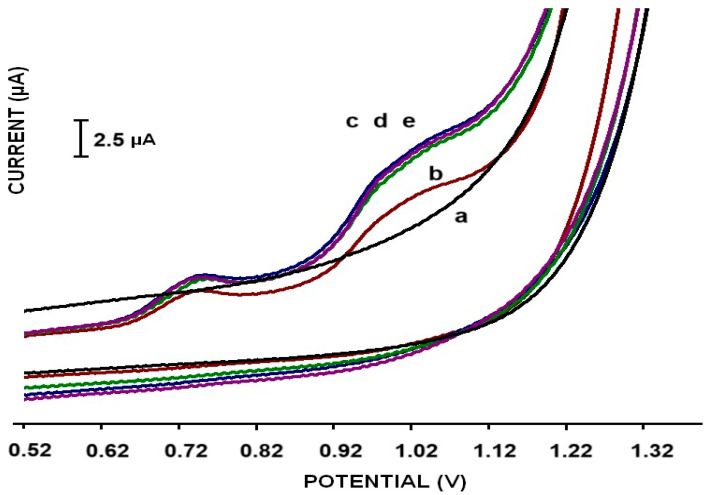
CV responses recorded from Mn-NPs nanoparticle-modified PGE surfaces at the solution phase in the absence and presence of 100 mg·L^−1^ dsDNA in PBS at a scan rate of 100 mV/s.

According to Figure 11, no peak was obtained around 1.0 V (guanine oxidation region) in the measurement taken only in PBS with the nanomaterial-modified electrode (Figure 11a). In the presence of 100 mg·L^−1^ dsDNA in the buffer solution, b, c, d and e signals were obtained, respectively. Of these signals, b; P1, c; P2, d; and P3, e; were obtained from the P4-type nanomaterial-modified electrode. Although dsDNA signals were obtained with all nanomaterial-modified sensors, similarly to the experiments performed on the surface phase (Figure 7 and Figure 8), a lower guanine response was obtained with the electrode containing the P1 nanomaterial at 1.37 μA (Figure 11b) compared to the others (Figure 11c–e, 1.80 μA, 1.70 μA, 1.98 μA, respectively). However, in line with the results obtained, it is proven that dsDNA determination can be made with all four types of nanomaterials [47]. More specifically, it was determined that P1 provides a more “ssDNA friendly” environment due to having NH_2_ groups from oleylamine, while P2 and P4 provide a more “dsDNA friendly environment” due to having -OH groups from the coating of triethyleneglycol molecules, with similar properties exhibited for solely P4. Yet, the same properties are indicated for the naked MnCO_3_, and under this venue composition, the effect of the metal core cannot be excluded.

#### 4.5.6. Analytical Application in Human DNA

Analytically applied human DNA from a healthy volunteer was obtained following written informed consent and was selected as a real sample for analysis by the proposed method using the standard addition method. A working solution was prepared by taking an aliquot of dsDNA extract in the Tris–EDTA buffer and diluting it with a 0.20 M acetate buffer, pH 5.0. Then, an appropriate amount of this diluted solution was transferred to a voltammetric cell and the voltammetric procedure was conducted. According to the results, the amount of dsDNA extract was found to be 0.62 ± 0.03 mg·L^−1^ applying the MnO_2_/Mn_2_O_3_@TEG type of Mn-NP-modified biosensor.

## 5. Conclusions

The construction of a sensitive and reproducible electrochemical DNA biosensor by Mn-based NPs was explored. In that vein, through a polyol solvothermal process, organic coated Mn-based NPs have been newly prepared and characterized. Meanwhile, a label-free, simple, direct and sensitive electrochemical DNA biosensor based on a disposable pencil graphite electrode was designed.

In the literature, up until now, no study has used such a simple and direct voltammetric technique based upon modification with novel manganese nanoparticles. This fact is also supported by the cited articles which appeared in a recent review article published by our research group pointing out the benefits arising from nanotechnology; it gives researchers new ideas for integrating these technologies into devices that can be used anywhere at any time [48]. Various electrode geometries featuring microcavities can improve the electrocatalytic performance of these materials, such as by employing materials with tunable porosity [49] and self-rolled three-dimensional microcavities [50]. These recent technologies hold significant promise for the advancement of DNA sensors and biosensors, contributing to the greater relevance and impact of the current findings.

The composition and surface properties of synthesized Mn-NPs proved to possess unique characteristics for their application in the development of DNA biosensors.

More specifically, the main advantage of the designed method is the Mn-NPs electrode surface modification, and the single-use pencil graphite electrode DNA nano-biosensor provides an enriched electrochemical response with higher sensitivity compared to the bare (Mn-NP-free) PGE. Another advantage of the developed technique is that it can measure the internal guanine oxidation signal observed at approximately +1.0 V without the need for any other indicator.

In this study, both ssDNA and dsDNA analyses were also realized with the Mn-NP-modified electrode, and for the first time, the affinities of nanomaterial-modified surfaces of ssDNA or dsDNA were evaluated regarding good reproducibility with a nearly 17 min analysis time at 50 µL of the sample volume.

This is the first voltammetric study investigating the signal-enhancing effect of Mn-NPs on DNA analysis, which has shown that nanomaterial modification provides a significant increase in the guanine signal in line with the increase in the amount of DNA bound to the surface. In addition, it was determined that P1 (MnCO_3_@OAm) provides more “ssDNA friendly” environments and P2 (MnCO_3_@TEG), P3 (MnCO_3_) and P4 (MnO_2_/Mn_2_O_3_@TEG) provide more “dsDNA friendly” environments thanks to their structures and the properties of the groups they carry, and this will bring a new approach to DNA biosensor designs.

However, in agreement with the results obtained, it is proven that dsDNA and ssDNA determination can be made with all four types of nanomaterials.

## Figures and Tables

**Table 1:** Calculated electroactive surface area (ESA) values (n = 4) obtained using Mn-NP-modified and -unmodified PGE by using 50 mV/s scan rate values.

A (cm^2^)	Type of PGE Surface
0.092	**Bare PGE**
0.128	**P1-mod. PGE** (MnCO_3_@OAm)
0.143	**P2-mod. PGE** (MnCO_3_@TEG)
0.131	**P3-mod. PGE** (MnCO_3_)
0.147	**P4-mod. PGE** (MnO_2_/Mn_2_O_3_@TEG)

## Data Availability

Data will be made available on request.

## Supplementary Materials

The following supporting information can be downloaded at: https://www.mdpi.com/article/10.3390/mi16020232/s1, Figure S1: Electrochemical impedance graphs including adjustments made by the equivalent circuit for impedance measurements made with Galvanoplot USB-type potentiostat device. Bare PGE (light blue line) and Mn-NPs- modificated PGEs as P1-PGE (blue line), (c) P2-PGE (red line), (d) P3-PGE (light pink line), (e) P4-PGE (light green line); Figure S2: Cyclic voltammograms of (A) bare PGE, and (B) P1 modified PGE (C) P2 modified PGE (D) P3 modified PGE, (E) P4 modified PGE at different scan rates of 10–300 mV s^−1^ in 5 mM K_4_[Fe(CN)_6_]/K_3_[Fe(CN)_6_] (containing 0.1 M KCl); Figure S3: A. SEM images of Mn-NPs modified electrode surface (40 µm), B. SEM images of Mn-NPs modified electrode surface (10 µm); Figure S4: SWV signals for the determination of human DNA isolated from a healthy volunteer at MnO_2_/Mn_2_O_3_@TEG modified electrode surface; Two standard additions of 0.60 mg L^−1^ dsDNA.
