# Supplementary material for: Nanoparticle-Based DNA Biosensor: Synthesis of Novel Manganese Nanoparticles Applied in the Development of a Sensitive Electrochemical Double-Stranded/Single-Stranded DNA Biosensor
Source: Micromachines (Basel). 2025 Feb 18;16(2):232. doi: 10.3390/mi16020232 (PMC11857743; doi:10.3390/mi16020232)
Supplement: Supplementary file 1 [file micromachines-16-00232-s001.zip › micromachines-3448665-supplementary.pdf]

# Supporting Information

# Nanoparticle Based DNA Biosensor; Synthesis of Novel Manganese Nanoparticles Applied in the Development of a Sensitive Electrochemical ds/ssDNA Biosensor

**Dilsat Ozkan-Ariksoysal [1,*], Elpida Pantelidou [2], Catherine Dendrinou-Samara [2] and Stella Girousi [3,*]**

[1] Department of Analytical Chemistry, Faculty of Pharmacy, Ege University, 35100 Bornova-Izmir, Türkiye
[2] Laboratory of Inorganic Chemistry, School of Chemistry, Aristotle University of Thessaloniki, 541 24 Thessaloniki, Greece
[3] Laboratory of Analytical Chemistry, School of Chemistry, Aristotle University of Thessaloniki, 541 24 Thessaloniki, Greece
* Correspondence: dilsat.ariksoysal@ege.edu.tr (D.O.-A.); girousi@chem.auth.gr (S.G.)

## 4. Results and Discussion

### *4.3. Electrochemical Characterization of the Mn-based Nanomaterials*

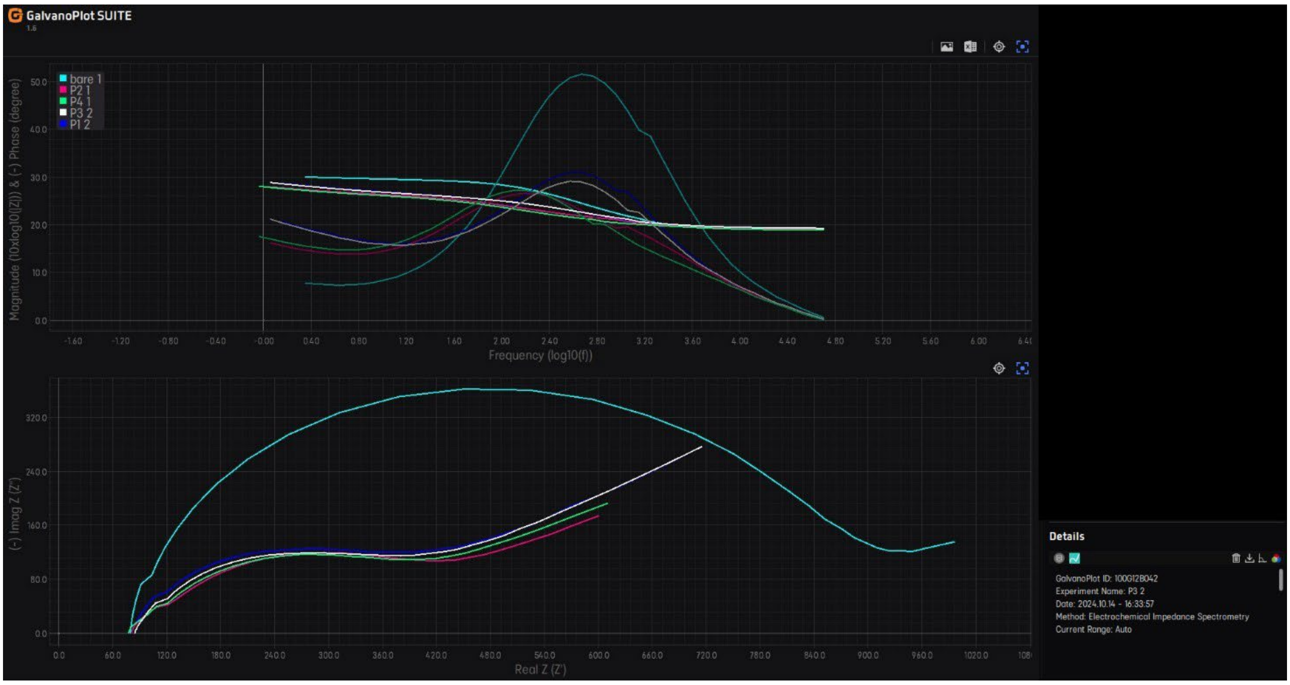


**Figure S1.** Electrochemical impedance graphs including adjustments made by the equivalent circuit for impedance measurements made with Galvanoplot USB-type potentiostat device. Bare PGE (light blue line) and Mn-NPs-modificated PGEs as P1-PGE (blue line), (c) P2-PGE (red line), (d) P3-PGE (light pink line), (e) P4-PGE (light green line).

*4.4. The Effect of Scan Rate on the Ferricyanide Signal*

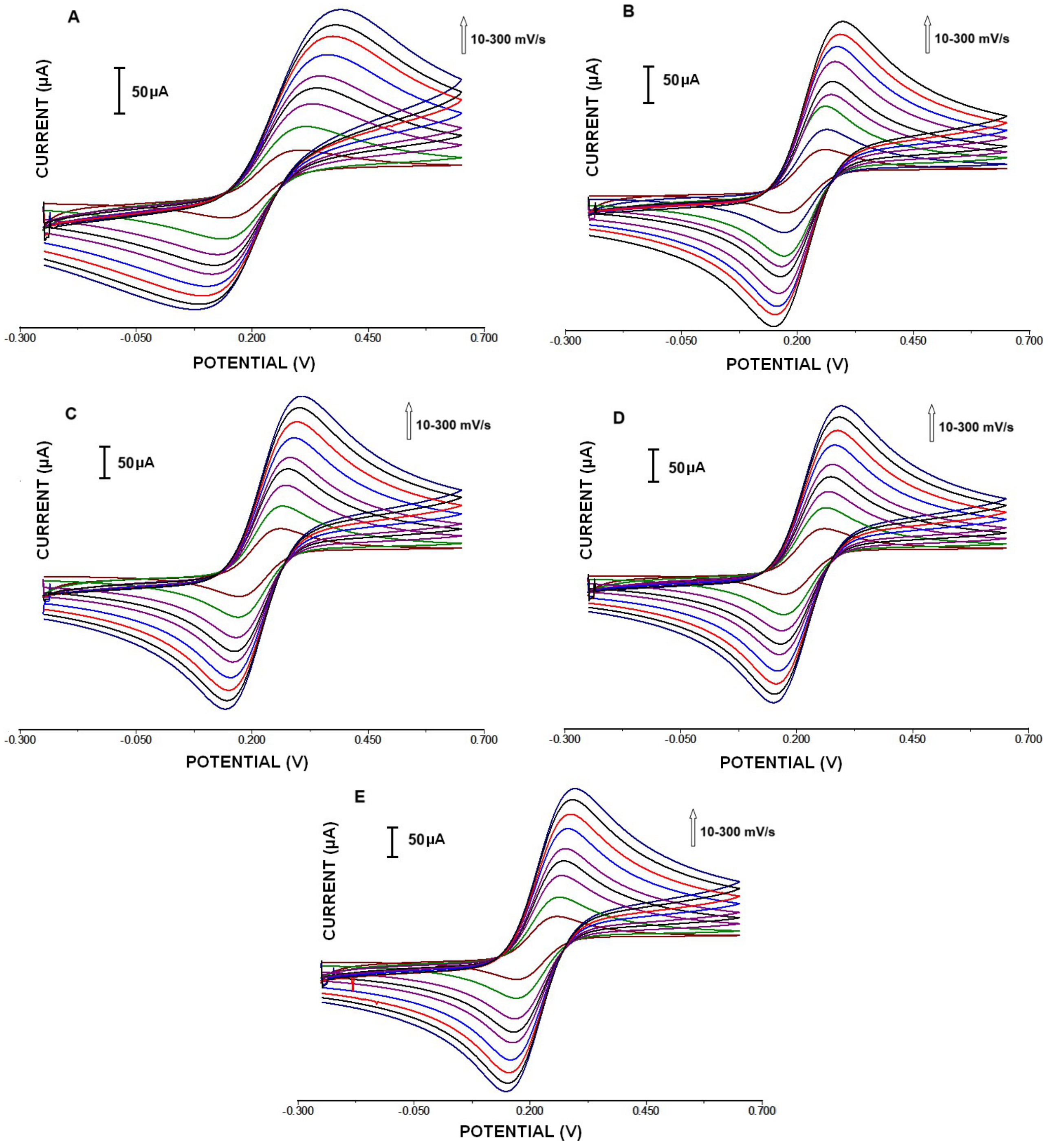


**Figure S2.** Cyclic voltammograms of (A) bare PGE, and (B) P1 modified PGE (C) P2 modified PGE (D) P3 modified PGE, (E) P4 modified PGE at different scan rates of 10 - 300 mV $s^{-1}$ in 5 mM $K_4[Fe(CN)_6]/K_3[Fe(CN)_6]$ (containing 0.1 M KCl).

*The characterization of Mn-NPs modified PGEs by scanning electron microscopy (SEM)*

In this study, the surface morphologies of pencil-tip graphite electrodes modified with Mn-NPs were examined using scanning electron microscope (SEM) at 2500 magnification rate and 40 μm sizes (Figure 3S-A) and 10000 magnification and 10 μm sizes (Figure 3S-B), respectively. Bare (unmodified), P1, P2, P3 and P4 modified PGE are presented in the images.

**Figure S3A.** SEM images of Mn-NPs modified electrode surface (40 μm)

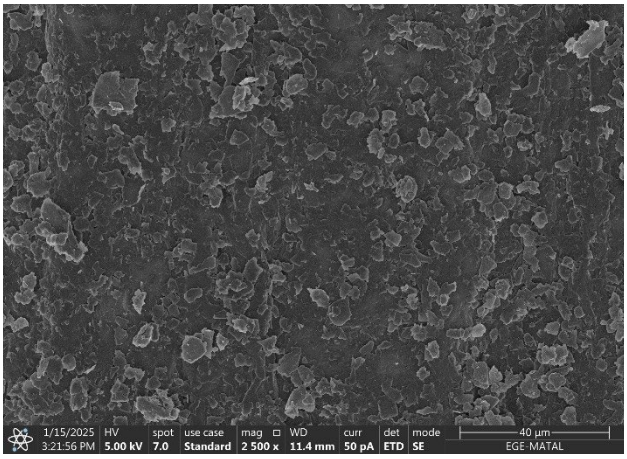


Bare

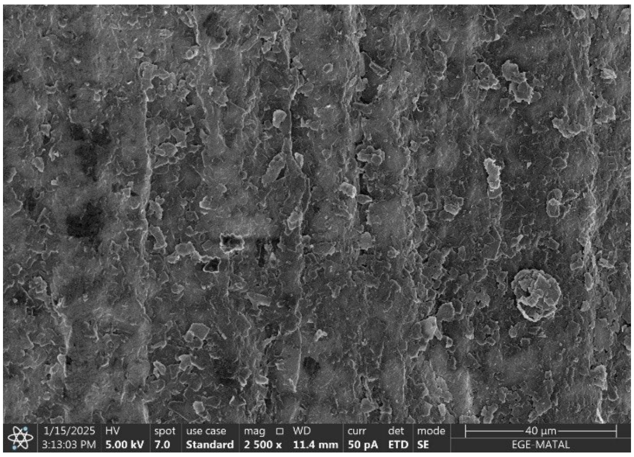


P1 modified

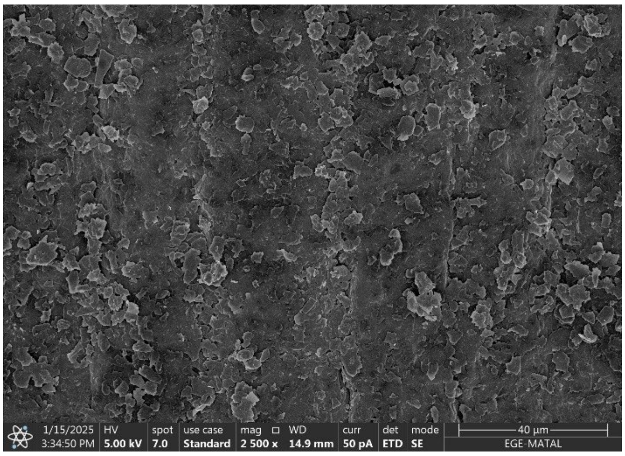


P2 modified

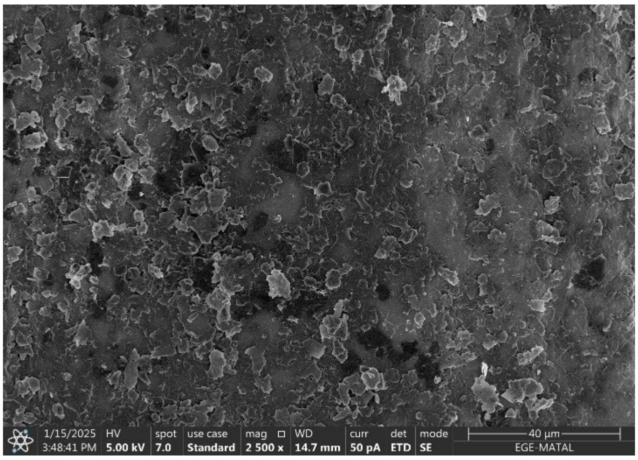


P3 modified

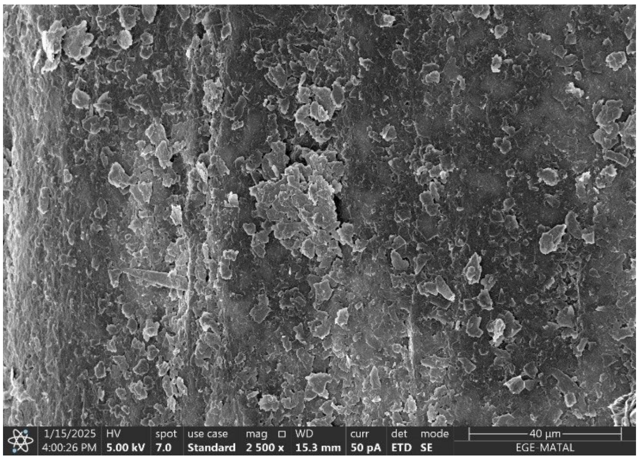


P4 modified

**Figure S3B**. SEM images of Mn-NPs modified electrode surface (10 μm)

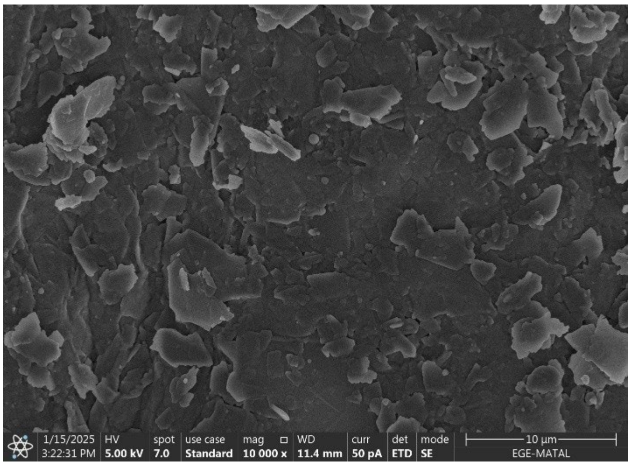


Bare

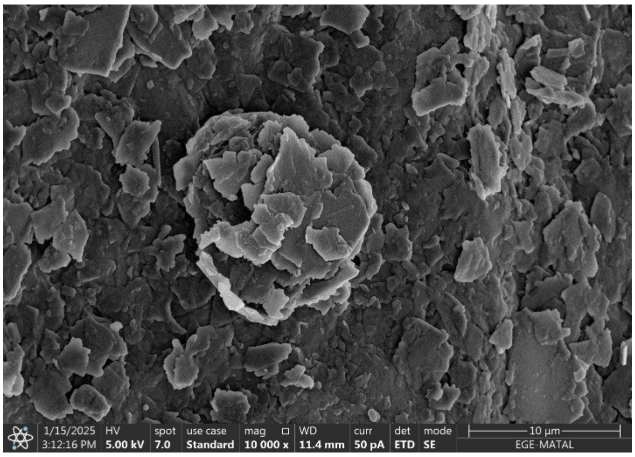


P1 modified

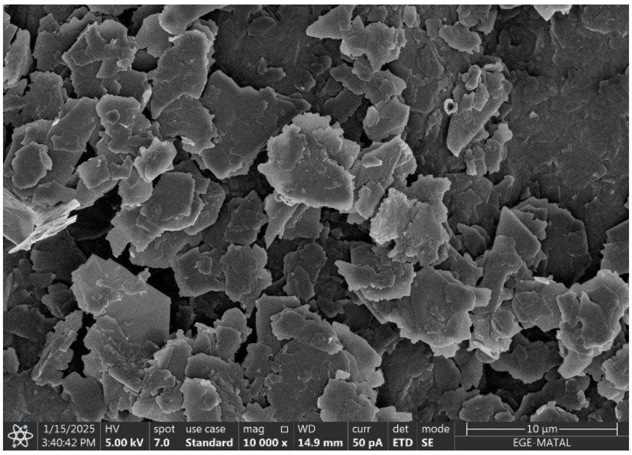


P2 modified

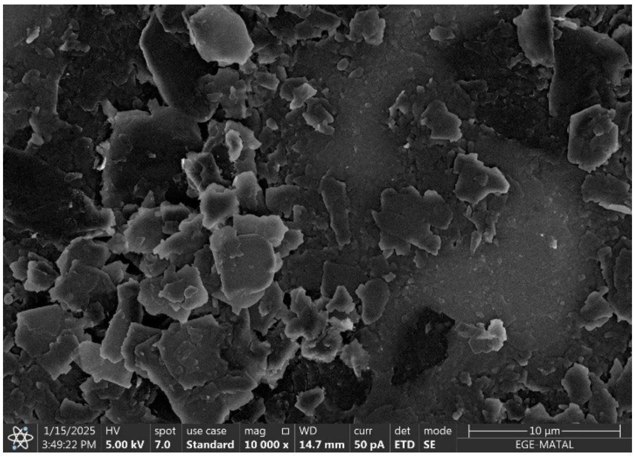


P3 modified

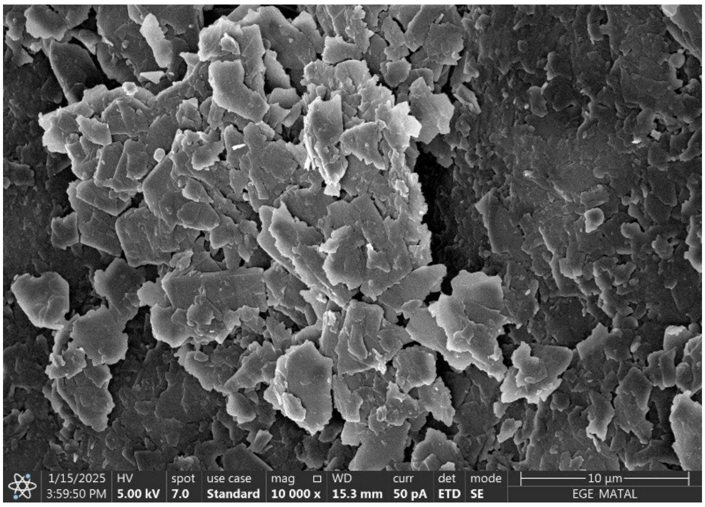


P4 modified

It was observed that the unmodified electrode had a surface structure containing planar graphite layers. The SEM image obtained after the P1 type Mn-NPs modification on the PGE surface showed that this nanomaterial caused an increase in surface roughness due to its different structure compared to the bare electrode, with clusters forming a flower-like appearance. It was evaluated that these regular and well-

defined clusters on the PGE surface increased the electrode surface area and thus the modification with P1 type Mn-NPs enhanced the electrochemical signal by binding dsDNA to PGE in greater amounts.

The SEM images obtained from the PGE surface modified with P2 type Mn-NPs, unlike the bare PGE, contain distinct layer structures that are clustered. This clustering is spread over the entire surface and has a homogeneous structure, almost covering the surface.

In the image obtained from the PGE modified with Mn-NPs P3, it is seen that there is no complete modification (coating) on the surface, but there are limited number of clustered layers. These images also show dark colored oval-shaped particles with well-defined boundaries of micrometer size.

The P4 type modified electrode with Mn-NPs showed a different morphology where these Mn-based nanoparticles were slightly but clearly clustered. However, when compared with the surface appearance obtained from the P2 modified electrode by SEM, there is a widely distributed clustered layering in P2, while there are layer clusters that do not completely spread on the surface in the P4 modified PGE. In other words, it is observed that the Mn-NPs are attached to the PGE surface, but they are clustered/grouped in clusters.

It was evaluated that there was a possible increase in the area of the modified PGE surfaces with the Mn-NPs of the types P1, P2, P3 and P4, whose surface morphologies were explained above, and as a result, the dsDNA bound more to the PGE with the modification process. It was concluded that the optical measurement results supported the findings provided by the electrochemical characterization. This shows that the more sensitive analysis related to ss or dsDNA detection can be done on the modified PGE surfaces with Mn-NPs nanoparticles.

4.5.6. Analytical Application in human DNA

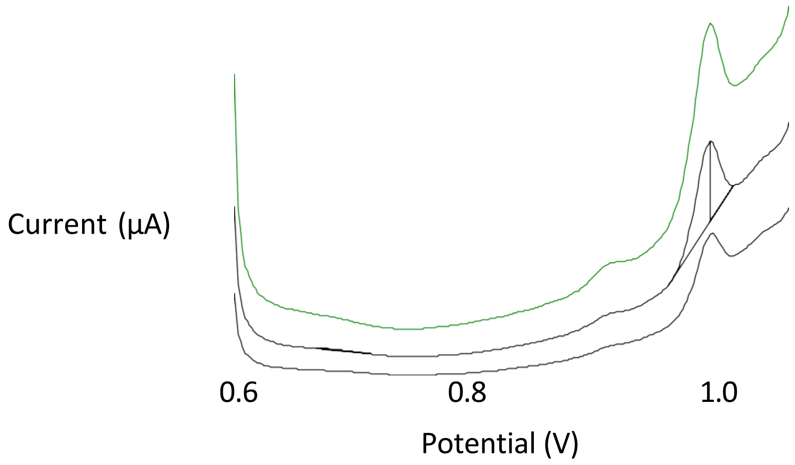


**Figure S4.** SWV signals for the determination of human DNA isolated from a healthy volunteer at $MnO_2/Mn_2O_3$@TEG modified electrode surface; Two standard additions of 0.60 mg $L^{-1}$ dsDNA
